# Current Advances in Nanomaterials Affecting Functions and Morphology of Platelets

**DOI:** 10.3390/jfb15070188

**Published:** 2024-07-08

**Authors:** Dongxin Peng, Sujing Sun, Man Zhao, Linsheng Zhan, Xiaohui Wang

**Affiliations:** Institute of Health Service and Transfusion Medicine, Beijing 100850, China; jya980104@163.com (D.P.); ammsreb@163.com (S.S.); zhaoman010@126.com (M.Z.)

**Keywords:** nanoparticles, platelets, interaction, activation

## Abstract

Nanomaterials have been extensively used in the biomedical field due to their unique physical and chemical properties. They promise wide applications in the diagnosis, prevention, and treatment of diseases. Nanodrugs are generally transported to target tissues or organs by coupling targeting molecules or enhanced permeability and retention effect (EPR) passively. As intravenous injection is the most common means of administration of nanomedicine, the transport process inevitably involves the interactions between nanoparticles (NPs) and blood cells. Platelets are known to not only play a critical role in normal coagulation by performing adhesion, aggregation, release, and contraction functions, but also be associated with pathological thrombosis, tumor metastasis, inflammation, and immune reactions, making it necessary to investigate the effects of NPs on platelet function during transport, particularly the way in which their physical and chemical properties determine their interaction with platelets and the underlying mechanisms by which they activate and induce platelet aggregation. However, such data are lacking. This review is intended to summarize the effects of NPs on platelet activation, aggregation, release, and apoptosis, as well as their effects on membrane proteins and morphology in order to shed light on such key issues as how to reduce their adverse reactions in the blood system, which should be taken into consideration in NP engineering.

## 1. Introduction

Over the past few years, nanotechnology has been developing rapidly, and nanomaterials have shown a wide range of applications in the field of biomedical science, including drug delivery, biological imaging, and tumor treatment. Nanomaterials can be categorized into organic and inorganic types. Organic nanomaterials encompass lipid nanoparticles (NPs) (such as liposomes) and polymer NPs (such as chitosan). Inorganic nanomaterials include metal NPs (gold, silver, zinc oxide, etc.) and non-metallic nanomaterials (such as silica, carbon nanotubes, etc.). The physical and chemical properties of NPs, such as morphology, particle size, hardness, surface charge, and functional groups, vary widely. However, what they have in common is that most NPs enter the body along the venous route and work within the circulatory system, resulting in interactions between NPs and blood components. Furthermore, due to their small particle size, NPs can easily cross various physiological barriers and are distributed throughout the body before entering cells through various mechanisms such as macropinocytosis, caveolin-mediated endocytosis, and clathrin-mediated endocytosis [1,2]. The adverse effects of NPs on red blood cells in structure, function, and in vivo circulation time after entering the circulatory system have been investigated [3]. However, the physicochemical properties of platelets are complex, and NPs can influence their physiological function through various pathways, so that the effects vary depending on the size, type, and charges of NPs. Here we focus on the relationships between the physical and chemical properties of NPs and their biocompatibility with platelets in order to draw our own conclusions and facilitate the design of nanomedicines that are likely to reduce the toxic and side effects on organisms.

Although platelets are the smallest of the blood cells, they are among the active components that nanomedicines encounter early in the human circulatory system. Platelets are produced by megakaryocytes and have a length of 1.5~4 μm and a width of 0.5~2 μm. Some are oval or disc-shaped, while others have a rhombus or irregular shape. Platelets do not have a cell nucleus but contain cellular organelles, and their internal structure contains scattered granular components such as α granules and dense granules. The lifespan of platelets in the human body averages 7 to 14 days. Platelets are normally in a resting state, but they can be activated by physiological and pathological factors before aggregation, release, and adherence upon stimulation. Platelets can be stored in a platelet thermostatic oscillator at 22 °C for five to seven days [4]. However, the function and progressive activation of platelets can diminish during storage. It has been found that zinc oxide NPs can reduce the potential of thrombin production [5], while silver NPs possess an inherent anti-platelet property [6] that can delay platelet activation and aggregation, which may slow the release and apoptosis of platelets and prolong their ex vivo storage time. In this regard, they are potential novel coating materials for platelet bags. Microfluidic systems have also been used to simulate blood vessels and explore the effects of NPs on platelets inside the body in hopes of finding out more about the interactions between NPs and platelets [7]. In fact, when the interaction between the negative-charged platelets and NPs happens, not only would the surface charge of NPs likely change, but differences in the morphology and size of NPs would likely also be observed, especially in cases of adhesion between them.

On this base, we have reviewed the effects of various NPs on platelets. Our findings suggest that, on one hand, the blood compatibility of nanomaterials as drug carriers after entering the body is a real concern, and on the other hand, nanodrugs are increasingly used in the fields of anticoagulation and thrombolysis so that strategies should be adopted to screen for more active candidate drugs. This review will focus on some common NPs and explore the ways in which they interact with platelets and affect their physiological functions (Table 1). We hope that this review can provide some reference for the selection and design of nanocarriers and nanodrugs so that NP–blood compatibility and biological safety can be improved, adverse reactions caused by NPs minimized, and the preservation of platelet function maximized.

## 2. Influence of NPs on Platelets

Platelet membrane surface proteins include sialic acid, P-selectin, integrin GPIIb/IIIa, and GPVI. One of the most prevalent glycoproteins on platelet membranes is GPIIb/IIIa, a member of the integrin receptor family. It is made up of two glycoproteins, GPIIb and GPIIIIa, which are arranged in a 1:1 ratio on membranes. GPIIIb/IIIa can bind to various platelet ligands, including fibrinogen, fibronectin (Fn), and von Willebrand Factor (vWF). Sialic acid is widely distributed in platelets and various biological tissues. In the presence of a large number of carboxyl groups, sialic acid molecules present a negative charge. Under physiological conditions, platelets carry a negative charge, and electrostatic repulsive forces can prevent platelet aggregation. When activated, platelets have their surface sialic acid desialylated, thereby accelerating platelet aggregation and shortening their lifespan [17,18]. During participation of platelets in hemostasis, the important adhesion receptor GPVI on the platelet surface is activated by various endogenous or exogenous ligands. Through immunoreceptor tyrosine-activated motifs (ITAMs), phosphorylation signaling is transmitted to drive platelet activation and regulate various functions, including platelet adhesion and aggregation [19]. P-selectin (CD62P) is more highly expressed in activated platelets and activated endothelial cells. In a resting state, P-selectin is stored within platelet granules. When stimulated by external factors, P-selectin rapidly fuses with the cell membrane and is expressed on the cell membrane surface. Therefore, the expression of P-selectin is one of the molecular markers of platelet activation.

### 2.1. Impact of NPs on Platelet Membrane Proteins

**Figure 1:**
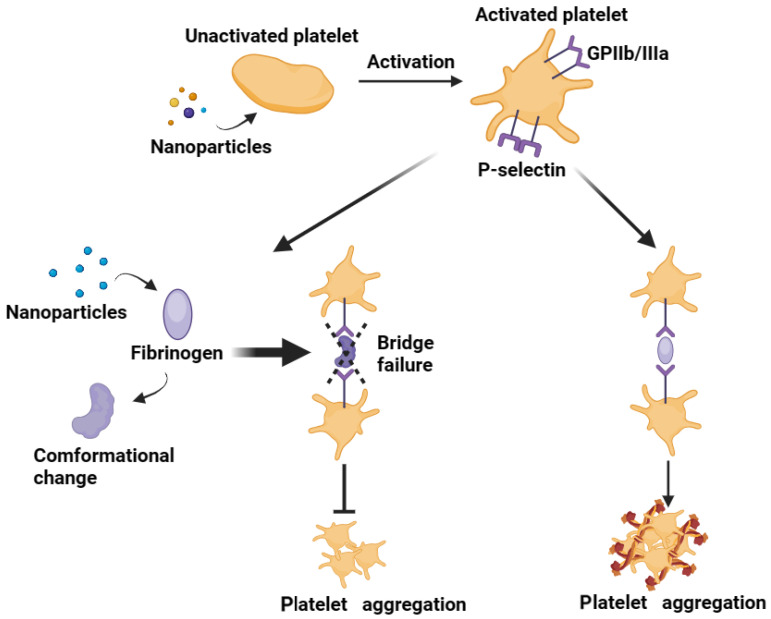
Some NPs (amorphous SiO_2_ [8,9], polystyrene [11], platinum [11], polystyrene latex [12] NPs) can interact with platelet, converting them from a non-activated state to an activated state. Simultaneously, the exposure of GPIIb/IIIa and the bridging effect of fibrinogen contribute to the aggregation of activated platelet. Additionally, other NPs (polyvinyl alcohol-coated superparamagnetic iron oxide [16] NPs) can affect fibrinogen, altering its conformation and preventing it from bridging platelets, thus reducing the aggregation of activated platelets. Figure 1 was drawn by the authors.

NPs can interact with numerous components of the circulatory blood, including platelets that are thus activated [20]. The interactions and reactions of NPs with platelets can be initiated by stimulating platelet surface receptors [21] or by disrupting the platelet membrane [22]. For instance, NPs interact with integrin GPIIb/IIIa on the surface of platelet membranes [11,12,16,21,23] that are consequently activated from a resting state. This process is regulated by the size, charge, concentration, and surface area of NPs. Amorphous SiO_2_ NPs (10 nm, 50 nm, 150 nm, and 500 nm) were found by Corbalan et al. [8] to induce the release of nitric oxide (NO) from platelets. This is followed by a massive stimulation of peroxynitrite (ONOO^−^), which results in an unfavorably low [NO]/[ONOO^−^] ratio, the exposure of GPIIb/IIIa on platelet membranes, and an upregulation of P-selectin expressions. These events ultimately cause platelet aggregation through pathways that are dependent on ADP and matrix metalloproteinase-2 (MMP-2). Among them, small-sized (10 nm) amorphous SiO_2_ NPs aggregate most quickly by interacting with platelet membrane proteins. To summarize, the impact of amorphous SiO_2_ NPs on platelet aggregation is inversely correlated with their size. This is also supported by the research conducted by Zia and colleagues [11], who found that different-sized polystyrene (25, 50, 119, 151, 201 nm) and platinum (7, 73 nm) NPs induce GPIIIb/IIIa exposure through passive aggregation, which is then regulated by Src and Syk tyrosine kinases, ultimately leading to platelet activation. The intensity of aggregation is also negatively correlated with particle size. In addition, the surface charge and concentration of NPs also affect platelet activation and aggregation. Smyth and others [12] used polystyrene latex NPs (50, 100 nm; PLNPs) modified by different functional groups. Although most of the tested PLNPs induced GPIIIb-/IIIa-mediated platelet aggregation, the intensity is related to physical interactions between PLNPs and platelet membranes or endocytosis of PLNPs. At a concentration of 50 nM, amine-modified PLNP (aPLNP) is more likely to cause platelet aggregation than carboxyl-modified PLNP (cPLNP) by means of physical bridging with adjacent non-activated platelets. Polyvinyl alcohol (PVA)-coated superparamagnetic iron oxide NPs (PVA-SPIONs), measuring 78 ± 22 nm in size, demonstrated dose-dependent anti-platelet action, according to research by Kottana and colleagues [16]. Platelet aggregation is prevented in the presence of PVA-SPIONs because the structure of fibrinogen is changed and bridge connections between platelets fail (Figure 1). This inhibitory effect is more pronounced at higher concentrations (250, 500 μg mL^−1^). The platelet membrane surface receptor P-selectin can be used for the design of targeted drug delivery. For instance, Cao et al. [24] created an NP complex called TM33-GON/TNA, which is modified by the TM33 peptide and loaded with tanshinone IIA (TNA). This NP complex can bind to the activated platelet surface specifically through P-selectin and release TNA into the extracellular space when matrix metalloproteinase-2 (MMP-2) is stimulated, resulting in high local TNA exposure. By inhibiting the conversion of resting platelets to activated ones, the tumor vascular endothelial barrier is disrupted. Consequently, platelet activation, adhesion, and aggregation around activated platelets are effectively inhibited, resulting in tumor endothelial leakage and enhanced tumor treatment efficacy.

Platelet microparticles (PMPs) are ultra-micro membranous vesicles released by platelets during activation. Their diameter is less than 0.5 μm, they have strong procoagulant activity, and they play an important role in the process of human thrombosis and hemostasis. The coagulation function of PMPs is attributed to the interactions between coagulation factors (primarily factors VII, IX, X, and prothrombin) and the negatively charged phosphatidylserine on the surface of PMPs [25]. Although platelets cannot cross tissue barriers, their extracellular vesicles (EVs) can enter lymph, bone marrow, and synovial fluid, which allows for the transfer of platelet-derived content to cellular recipients and organs inaccessible to platelets. Looking beyond hemostasis, PMP cargo is incredibly diverse and can include lipids, proteins, nucleic acids, and organelles involved in numerous other biological processes [26]. This versatility expands the application of PMPs in other physiological and pathological aspects besides hemostasis. In addition, platelets also possess the ability to phagocytose viruses, bacteria, and other particles, and can even engulf NPs. For instance, activated platelets can internalize inert latex microspheres (MS, 200 nm) without affecting blood platelet aggregation [27], providing an ideal carrier for a platelet-based drug delivery system. Electron microscopy results demonstrate that small-sized silver NPs (13, 20, 29 nm) interact with the platelet membrane and accumulate in platelet granules, effectively inhibiting integrin-mediated blood platelet aggregation in a concentration-dependent manner both in vitro and in vivo [6]. Differently designed platelet-based platforms can be constructed for drug administration by taking advantage of the binding and engulfment between NPs and platelets, as well as the targeting property of platelets directly.

When nanomedicines enter the circulatory system, proteins can be spontaneously adsorbed onto the NP surface to form a protein corona [28]. For example, albumin can form a protein corona around NPs [29]. The uptake of NPs into cells and their aggregation in cell culture can both be decreased by albumin adsorption. This has an impact on how NPs interact with biological systems, changing their toxicity and distribution and improving their biocompatibility [30]. In order to study the function of platelet transfusion in vivo, researchers reported a method of modifying the NP surface with human serum albumin (HSA) to improve the uptake of platelets containing Resovist^®^ (an FDA-approved MRI contrast agent) and reduce the pre-activation of platelets in response to NPs. This approach improved the bio-absorption and toxicity of NPs in platelet labeling and allowed for the recovery of labeled platelets from whole blood using magnetic separation [31]. Additionally, there are reports that perfluorotributylamine NPs (150, 200 nm, PFTBA) have platelet-inhibitory effects. Perfluorotributylamine NPs (PFTBA@Alb) are created when albumin is utilized as the shell. This results in NPs with an improved platelet-inhibitory activity that successfully increases the permeability of tumor blood arteries without having a substantial negative impact on normal capillaries. This approach can effectively increase the permeability of tumor blood vessels by inhibiting platelet function, promoting the infiltration of immune cells into tumors, and thereby significantly enhancing the therapeutic effects of tumors [15].

NPs can also interact with endothelial cells. Studies conducted by Saikia and colleagues [32] have demonstrated that the presence of silica NPs in the venous system activates endothelial platelet adhesion receptors, resulting in an increased number of platelets adhering to the endothelial cell surface. One possible explanation is that NPs promote the development of adhesion molecules linked to inflammatory indicators, such as VCAM-1, ICAM-1, and PECAM-1, and produce oxidative stress. These actions also alter the NO/ NO synthase (NOS) cycle. Ultimately, these factors impact the adhesion between platelets and endothelial cells. NPs can also lead to the overexpression of von Willebrand factor (vWF) polyproteins, which is a key step in the damage caused by platelet activation and NP adhesion [32,33].

NPs can impact platelets either directly or indirectly by inducing changes in membrane proteins, which in turn affects their functional status. Consequently, it is crucial to comprehensively grasp the inherent characteristics of NPs, as well as their interactions with platelets, and to understand the variations that arise from differences in size, charge, and concentration, which play a key role in enhancing the biocompatibility of NPs. Furthermore, by considering the unique properties of NPs, we can strategically select and design NP for specific applications under various scenarios.

### 2.2. Influence of NPs on Morphology of Platelets

The microscopic morphology of platelets can be examined by various technological means, such as transmission electron microscopy (TEM) and scanning electron microscopy (SEM). When NPs interact with platelets, they cause the contraction of the microtubule annular band and actin skeleton, resulting in the compression of cellular particles towards the central part of the cell. This leads to the fusion of particle surface or membrane with open tubular membranes and the extrusion of particle contents to the cell exterior through the open tubular structure [13]. Consequently, platelets are activated and morphological changes take place. Lipid NPs (LNPs) are crucial to lipid-based carrier drug delivery systems in which they can bind with small molecule drugs to achieve enhanced therapeutic effects [34]. The remodeling of platelet membranes is associated with the maturation of glycosylation in secreted platelet extracellular vesicles (PL-EVs) and B-type I scavenger receptor (SR-B1), initiating the transition from non-glycosylated to glycosylated forms. Mildly oxidized high-density lipoprotein (MoxHDL) remodels itself into corresponding phospholipids by enhancing platelet uptake of hemolytic phospholipids, thereby improving the lipid homeostasis of platelet membranes [35]. Similarly, sphingomyelin (SM) and sphingosine-1-phosphate (S1P) can also produce comparable effects [36], thus enhancing the quality and storage potential of platelets. Consequently, liposome NPs prepared by loading of small functional molecule substances into liposomes were expected to be used in platelet storage and prolong the storage time of blood products. In the presence of silver NPs, platelet adhesion can be prevented, and integrin-mediated platelet reactions can be effectively inhibited in a concentration-dependent manner. Fluorescence microscopy observation revealed that at a concentration of 5 μM nanoscale silver, the inhibitory effect was most pronounced, with minimal re-organization of F-actin and changes in the cell skeleton [37]. It is speculated that silver NPs may represent a novel strategy for maintaining a low activation state in platelets and preventing vascular thrombosis.

These insights open up avenues for research aimed at prolonging the morphological integrity, functional capacity, and lifespan of platelets, as well as advancing the design of innovative blood-storage bags.

### 2.3. NPs Influence Platelet Activation and Aggregation

Flow cytometry is an ideal technique for quantitatively detecting changes in the expressions of GPIIb/IIIa and P-selectin (CD62P) on platelets, which were the important markers that could be detected in platelet activation. This is achieved by discriminating platelets based on their size and granularity (forward and side scatter) and determining the expression abundance of the target by the fluorescence value of the antibody bound to it. Typically, light transmission aggregometry is used to identify platelet aggregation. Alternatively, dissipative quartz crystal microbalance (QCM-D) can be employed to characterize small aggregates [38].

When platelets are activated, the expression of P-selectin and GPIIb/IIIa (CD41/CD61) increases (Figure 1). Flow cytometry can be used to detect the positive rate on the surface of platelets [39,40]. Numerous studies have demonstrated that NPs can cause platelet activation, and that glycoprotein receptors may be essential to this process. Specifically, different G protein-coupled receptors mediate platelet-to-platelet contact and aggregation [41]. GPIIb/IIIa (CD41/CD61) is a pair of constitutive integrin receptors found in an inactive state on the surface of resting platelets, having a low affinity for adsorbed fibrinogen. In other words, GPIIb/IIIa induces or triggers platelet aggregation by using fibrinogen as a bridging molecule, thereby playing a pivotal role in primary hemostasis [42,43]. Furthermore, 20,000–40,000 receptors are present in the membranes lining the open tubular system within platelets, which are exposed on platelet membranes only upon stimulation by activating signals [44]. When NPs engage with platelets, they facilitate the exposure of additional integrins, bridge fibrinogen and surrounding resting platelets, and concurrently secrete cytokines, thereby accelerating platelet activation and aggregation [13]. During this process, one of the molecular markers of platelet activation, P-selectin, becomes exposed.

Research has demonstrated that NPs of different concentrations exert distinct effects on platelets. For instance, silver NPs inhibit platelet reactions in a concentration-dependent manner, with optimal inhibitory effects occurring at 5 μM for13–15 nm, 30–35 nm, and 40–45 nm sized ones [37]. Moreover, smaller NPs tend to have a more significant impact on coagulation than larger ones [13]. There have also been reports that contact between platelets and silver NP-coated catheters (characterized by a high surface area to volume ratio) can accelerate the production of thrombin, leading to enhanced platelet activation [45]. Titration experiments indicate that platelet activation may result from collisions with silver particles exposed on the surface, without adhesion to the surface [45]. These findings have boosted the development and improvement of indwelling catheters, prompting the exploration of new coatings with antimicrobial properties and the capacity to prevent thrombus formation. As a result, the procoagulant properties of NPs may increase risks for common diseases such as diabetes, cancer, and cardiovascular diseases. To mitigate the additional risks associated with NPs, Ragaseema et al. [14] prepared polyethylene glycol (PEG)-coated silver NPs (20 nm) (PEGeSNPs) on cardiovascular implant stents, which exhibit a direct inhibitory effect on platelet activation and aggregation. However, the application of this procoagulant effect is particularly urgent in severe local bleeding cases. Chung et al. [10] coated chitosan (CS) NPs with adenosine diphosphate (ADP) to accelerate platelet activation and then measured its hemostatic effect. Studies have found that these ADP-coated CSNPs can shorten the clotting time and produce stronger blood clots. Lord et al. [46] observed that platelet adhesion and activation induced by CS is significantly amplified in the presence of plasma proteins or extracellular matrix proteins. Furthermore, nanomaterials interact with platelets, increasing platelet activation and adhesion, accelerating platelet aggregation, and enhancing their hemostatic effects. Because of the significant antibacterial properties of chitosan and platelets against *Escherichia coli* and *Staphylococcus aureus*, their complex has been widely applied to wound dressings [47,48,49,50]. Numerous literature reports indicate that chitosan exhibits a broad-spectrum antimicrobial effect, capable of inhibiting various bacteria, fungi, and even some viruses. On the other hand, researchers have modified the surface of chitosan NPs with polyethylene glycol, polyvinyl alcohol, or ethylenediamine tetraacetic acid to reduce platelet aggregation or coagulation, which results in good blood compatibility [51].

As activation of integrin GPIIb/IIIa is one of the steps in the activation of platelets by numerous NPs [21,52], the inhibition of integrin-mediated platelet activation has become one of the crucial approaches to impeding platelet function. Among cancer patients, the efficacy of anticancer treatment is compromised due to the inability of drugs to penetrate the tumor vascular barrier. Albumin-coated perfluorocarbon NPs (PFTBA@Alb) were created by Zhou and colleagues [15] with the potential to non-specifically impair platelet function in tumors. This could cause the tumor vascular wall integrity to be disrupted, immune cell penetration into the tumor to be encouraged, and the anticancer effect to be enhanced. Furthermore, in the field of cancer treatment, it is a commonly used approach to inhibit platelet function to enhance drug penetration [53,54]. Arachidonic acid, under the action of platelet cyclooxygenase, produces prostaglandins, which play a significant role in promoting platelet aggregation. Another study found that silver NPs coated with polyethylene glycol (PEG) can inhibit the synthesis of arachidonic acid to thromboxane by affecting its metabolism, thereby blocking the exposure of GPIIb/IIIa and inhibiting platelet activation and aggregation. Besides antithrombotic properties, this polymer also possesses antimicrobial properties and good histocompatibility [14]. In addition, PEGylation during platelet storage is also a concern. PEG can react with lysine residues on the platelet surface to form a stable amide bond, thereby preventing the deformation and activation of platelet surface glycophorins during storage. PEGylation has also been employed in platelet storage to prevent bacterial contamination in blood bags and improve the safety of transfusion [55].

**Figure 2:**
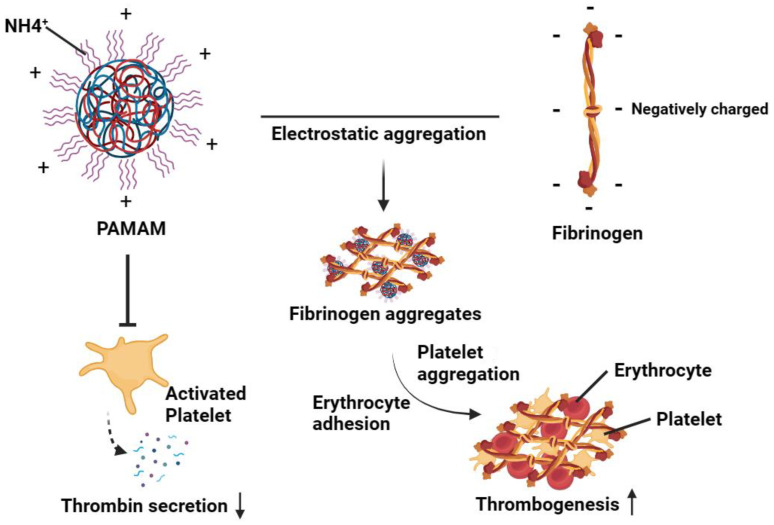
Dendritic macromolecules can reduce the production of platelet-dependent thrombin and promote platelet aggregation in a way that is independent of the classical clotting pathway. Fibrogen is with Negatively charged overall due to the presence of fibrin peptide A (FPA) and fibrin peptide B (FPB) monomers. The positively charged PAMAM interacts with the Negatively charged fibrinogen to form fibrinogen aggregates through electrostatic interaction. These aggregates promote platelet aggregation and erythrocyte adhesion, thereby increasing thrombus formation. Figure 2 was drawn by the authors.

Polyamidoamine dendrimers (PAMAM) have come to be one of the most extensively studied dendrimer architectures in recent years. Their surface is densely covered with functional groups that can be easily modified, and they possess a considerable number of cavities inside, making them an ideal choice for drug and gene delivery applications due to their excellent biocompatibility, permeability, and stability. However, research has also revealed that they can exhibit cytotoxicity, activate platelets, and significantly alter their morphology, leading to an increase in aggregation and adhesion [22]. A study conducted by Dobrovolskaia et al. [56] demonstrated that PAMAM dendrimers accelerated platelet aggregation by disrupting the integrity of platelet membranes. The property of PAMAM dendrimers is derived from the surface-active amine groups. Under physiological conditions, the amine groups at the ends are protonated to form positively charged ammonium ions, which may contribute to platelet activation and aggregation. This interaction is mediated through electrostatic interactions between the densely charged cationic dendrimer and anionic fibrinogen domain structures [57]. The degree of platelet aggregation is measured to be directly proportional to the number of amine groups. Further research has also shown that cationic PAMAM dendrimers can cause platelet dysfunction, such as reducing the production of platelet-dependent thrombin [22]. However, this does not essentially lessen hemostatic activity because they can act directly on fibrinogen without the help of thrombin, resulting in fibrinogen aggregates that are dense and have a high molecular weight, which increases the development of thrombi [57] (Figure 2). Unlike cationic PAMAM dendrimers, anionic PAMAM dendrimers with concentrations lower than 0.5 mg/mL usually do limited harm to platelets [58]. More research is needed to focus on the underlying mechanism in platelet activation and aggregation caused by PAMAM and to minimize its toxic effects in the blood. In recent years, PEG conjugation has become the most commonly used method to reduce the toxicity of surface active groups of dendrimers. Researchers have found that partially PEGylated PAMAM can reduce PAMAM polymer-mediated platelet activation and platelet reduction in vitro [59], thus increasing its biocompatibility and drug loading [60,61,62]. Research indicates that the charge, concentration [63,64], and size [65] of NPs can prolong the prothrombin time to varied extents [66]. Overall, small-sized, negative-charged, and PEGylated dendrimer NPs with low molecular weight and low concentration have advantages in improving the stability and survival rate of platelets during storage [55].

This empirical rule can provide some reference for exploring the relationship between the physical and chemical properties and biological effects of nanomaterials in terms of design, as well as improving drug distribution, accelerating circulation, and enhancing blood compatibility and biological safety.

### 2.4. Mechanisms of Activation and Aggregation Promoted by NPs

When NP-based adjuvants are introduced into organism, such exposure can trigger the activation of the NLRP3 inflammasome, which in turn promotes the secretion of inflammatory factors [67]. Similarly, silica NPs can induce inflammation by initiating the ROS/PARP-1/TRPM2 signaling pathway [68]. The elevated levels of these inflammatory factors within the body could result in platelet activation and aggregation, consequently raising the risk of thrombotic diseases [69].

Platelets are central to the development of thrombosis, performing a pivotal role in the formation of blood clots throughout the body. When nanomedicines enter the circulatory system, they interact with platelets either directly or indirectly, stimulating the activation of the coagulation cascade and the production of thrombin, which in turn activates platelets. Meanwhile, the shape of platelets changes, pseudopodia are extended, and 5-hydroxytryptamine and thromboxane A2 are released, causing vasoconstriction and platelet aggregation [70]. The exposure of integrin GPIIb/IIIa is one of the steps in the activation of platelets by various NPs [21,44,52]. After that, integrin GPIIb/IIIa emits a signal through the tyrosine-based sequence in the relevant membrane protein [11]. Platelet activation is caused by increased Ca^2+^ flow through pathways regulated by Src and Syk tyrosine kinase in vitro. Upon activation, the platelets undergo a conformational change, exposing the high-affinity binding site for soluble fibrinogen on GPIIb/IIIa [19], enhancing the interaction between fibrinogen and GPIIb/IIIa, and facilitating the formation of blood clots by binding to blood cells to accelerate the coagulation process. Besides platelets, NPs also engage with other blood components, such as plasma proteins (fibrinogen, coagulation factors, etc.) [71], and lead to complex changes in a multitude of blood physiological processes, including plasma protein adsorption, coagulation factor activation, platelet activation, and adhesion.

**Figure 3:**
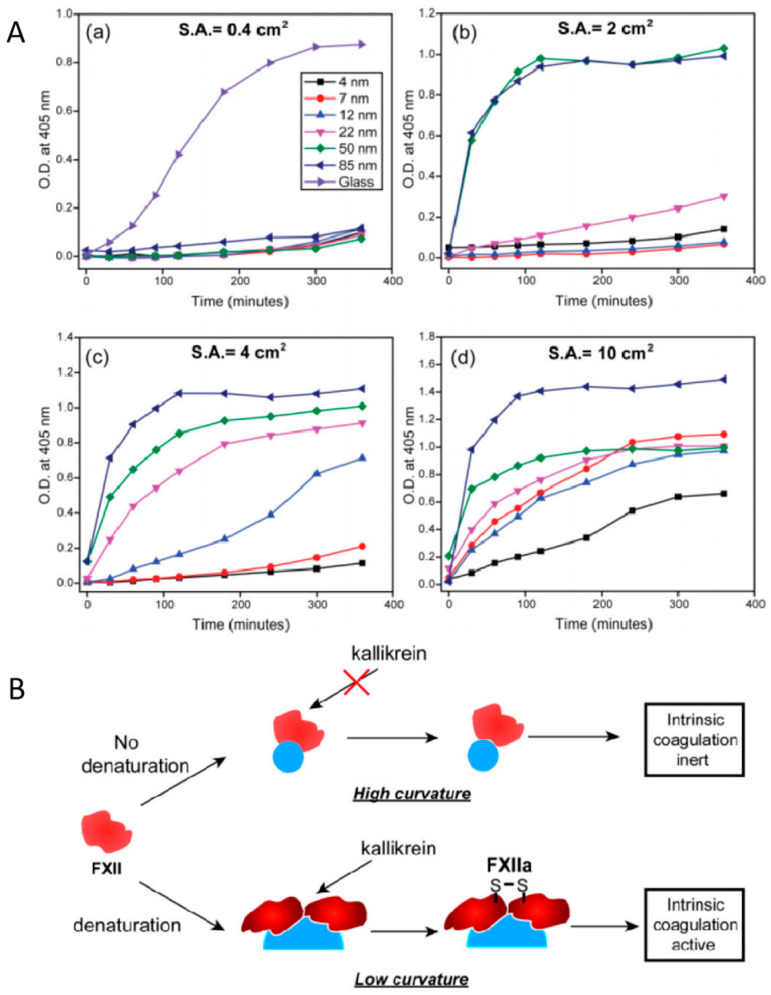
(**A**) Size-dependent intrinsic coagulation activity of silica NPs over time with surface area (S.A.) (**a**) 0.4 cm^2^, (**b**) 2 cm^2^, (**c**) 4 cm^2^, and (**d**) 10 cm^2^. Results are the averages of at least twelve replicates. O.D. denotes optical density. (**B**) Effect of NP surface curvature on intrinsic coagulation. NPs with higher surface curvature (smaller size) do not denature FXII on their surface and thereby are coagulation inert, whereas NPs with smaller curvature denature FXII, initiating coagulation cascade. Notes: copyright obtained from ref. [72]. The figure has been reproduced from ref. [72] with permission from the Royal Society of Chemistry, copyright 2023.

The physicochemical properties of nanomaterials can also be adjusted to regulate coagulation processes. It was found that SiO_2_ NPs activated the endogenous coagulation pathway, and the degree of activation increased with the specific surface area and silanol groups [9]. Another study reported that compared to high surface curvature (small size), low surface curvature (large size) had a greater degree of denaturing coagulation factor XII, resulting in higher coagulation activity in vitro (Figure 3A). Coagulation factor XII is activated by larger SiO_2_ particles instead of small-sized ones. It is speculated that larger particles may cause greater changes in the state of coagulation factors with blood clotting, and provides a basis for the treatment of traumatic injury (Figure 3B) [72]. In addition, when the body is stimulated or injured, platelets may release microparticles (MPs) with the size of about 100 nm and a lifespan of 10–30 min. As liposome NPs are derived from cells, the negatively charged phosphatidylserine on their surface interacts with coagulation factors in plasma (mainly factors VII, IX, X, and prothrombin) [25]. It is worth noting that these microparticles have a procoagulant activity 50–100 times higher than that of activated platelets under the same surface area [13].

By elucidating the molecular mechanisms behind NP-induced thrombosis, we gain new insights and potential therapeutic targets for the prevention and treatment of thrombotic disorders associated with nanomaterial exposure.

### 2.5. NPs Influence Platelet Release and Apoptosis

Upon activation, platelets release a multitude of molecules. The majority of these bioactive molecules are released from α-granules, which are unique to platelets and contain an incredibly diverse repertoire of cargo, including integral membrane proteins; pro-coagulant molecules; chemokines; mitogenic, growth, and angiogenic factors; adhesion proteins; and microbicidal proteins. The irreversible nature of α-granule secretion makes it ideally suited as a marker of platelet activation [73]. The process of secretion and release of dense alpha particles in platelets is called ‘release reaction’. Typically, NP tracking analysis (NTA) is used to analyze the release of platelet-derived vesicles (PEVs) [35]. Generally speaking, the activation of platelets depends on mitochondrial function [74], and the lifespan of most platelets is strictly regulated by mitochondrial apoptosis, mainly controlled by the BCL-2 protein family, which is crucial to maintaining platelet physiological function [75]. Triphenylphosphine compounds increase the exposure of phosphatidylserine on the platelet membrane surface, and a series of in vitro experiments have shown that they cause a decrease in mitochondrial potential, suggesting the impact of mitochondrial function on platelets [74]. Therefore, the changes in the mitochondrial membrane potential of platelets and the surface exposure of phosphatidylserine are important indicators for evaluating blood compatibility of NPs.

During the activation of platelets by NPs, membrane proteins such as GPIIb/IIIa and P-selectin, which mostly undergo conformational changes and migrate from the cytoplasm, are exposed on the cell surface and participate in the activation and aggregation of platelets. For instance, after activation, the affinity of surface glycoproteins on platelets for NPs increases, and their interaction is enhanced and characterized by elevated adhesion and aggregation and the release of cytokines. However, it remains unclear whether this process is connected to apoptosis. Research has shown that inhibiting the anti-apoptotic protein BCL-XL can lead to rapid platelet apoptosis, resulting in a decrease in platelet count [75]. Using Bcl-xL–inhibitory BH3 mimetics was shown by Schoenwaelder and others [76] to accelerate platelet apoptosis and downregulate integrin GPIIb/IIIa adhesion function, meaning that GPIb and GPVI surface expression is reduced, PS exposure is increased, aggregation is decreased, and blood-clotting ability is decreased. However, the question of whether the change in the expression level of the anti-apoptotic protein BCL-XL can be used as one of the indicators of platelet apoptosis promoted by NPs requires more research. Andelman and others observed that the biological compatibility of NPs with different microscopic morphologies differs. Compared to spherical and rod-shaped particles, nanosheets have much higher cytotoxicity [77]. Zinc oxide NPs induce various changes in cell parameters in platelets and other cells and cause mitochondrial membrane damages and the production of reactive oxygen species, resulting in cell toxicity and genotoxicity effects and accelerating cell apoptosis [78]. Some NPs may actually delay platelet apoptosis. For instance, mildly oxidized HDL (MoxHDL), a biological small molecule derived from the human liver, can enhance the lipid stability of platelet membranes by reducing the release of vesicles during storage [35]. Lipid NPs can also collaborate with small molecules as a more effective treatment of diseases [34]. Furthermore, small silver NPs can inhibit platelet activation and aggregation, slowing down degranulation [37]. This may also delay platelet apoptosis.

In this subsection, we encapsulate the effects and general mechanisms of NPs on platelet secretion and apoptosis, expanding the choices available in the utilization of nanomaterials. The elucidation of the molecular pathways through which NPs trigger the release and apoptosis of platelets offers novel targets for the prevention and therapeutic intervention of nanomaterial-induced platelet-related diseases.

## 3. NPs in Anticoagulation and Anti-Thrombosis Applications

Thromboembolic conditions were estimated to account for one in four deaths worldwide and are the leading cause of mortality [79]. Platelets play a central role in thrombus formation, and therefore, it is of great importance to regulate and maintain platelet homeostasis and keep them in an inactive state. Clinical experts consider this the primary treatment goal for thrombotic diseases [80]. Researchers have found that silver NPs effectively inhibit integrin-mediated platelet functional responses in a dose-dependent manner, such as adhesion to fibrinogen and changes in the platelet cell cytoskeleton, significantly inhibiting platelet coagulation function [37]. Salidroside can inhibit platelet activation through the SIRT1/ROS/mtDNA pathway without increasing the risk of bleeding, thereby preventing thrombus formation [81], which offers hope for its application as an anti-platelet agent. As lipid NPs can be employed as a drug carrier of small molecules [34], it is worthwhile to construct engineered liposomes by encapsulating salidroside in them to increase biocompatibility, targeted delivery, and therapeutic effects.

**Figure 4:**
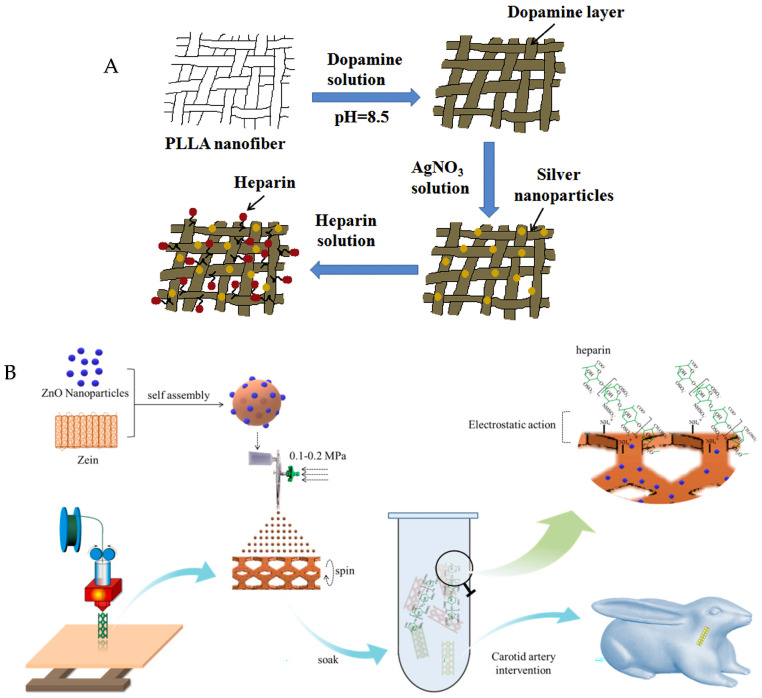
(**A**) Schematic illustration of the preparation process of PLLA—PDA, PLLA—PDA-Hep, PLLA—PDA—Ag, and PLLA—PDA—Ag—Hep fiber mats. (**B**) Fabrication process of multi-functional artificial conduits. Notes: copyright obtained from refs. [83,84]. (**A**) has been reproduced from ref. [83] with permission from Elsevier, copyright 2023. (**B**) has been reproduced from ref. [84] with permission from the Elsevier, copyright 2023.

Some clinical diseases lead to secondary platelet activation, which, in turn, activates surrounding resting platelets, thus forming multicellular aggregates connected by fibrin bridges and resulting in thrombus formation [82]. Platelet activation is a precursor to platelet aggregation. Generally speaking, the activation state of the platelet cannot be changed. However, Kottana and others have found that PVA-SPIONs affect the structure of fibrinogen and prevent the bridging between platelets and fibrinogen, thereby slowing down thrombus formation and inhibiting platelet aggregation [16]. Poly-L-lactic acid fiber mats, prepared by electrospinning and modified with poly-dopamine (PDA) and silver NPs (AgNPs) on the surface, provide a dual-function antibacterial/anticoagulant coating, offering hope for the development of a novel platelet blood bag (Figure 4A) [83]. Wang et al. combined 3D printing and coating techniques that encapsulated ZnO NPs in corn alcohol soluble protein NPs and enabled heparin to be adsorbed onto the surface to combat thrombus formation and postoperative infection caused by stent implantation (Figure 4B) [84].

## 4. Summary and Outlook

Nanomedicine has long been used in the field of modern medicine. NPs can serve as carriers or nanocoatings that enhance drug delivery efficiency or improve circulation cycles. Additionally, they can act as nanodrugs to treat various diseases due to their inherent properties. For instance, NPs have been applied in the fields of anticoagulation and antibacterial therapy by promoting platelet activation and aggregation, mediating thrombus formation, and achieving faster hemostasis.

The field of nanomedicine has been garnering increasing attention, and the blood compatibility of nanomaterials is a significant concern for researchers. Numerous reports have indicated that NPs can facilitate platelet activation, induce morphological changes, and generate a large number of pseudopodia, alter their shape, and enhance their cytoskeletal rearrangement. However, it remains unclear whether NPs will impact platelet transendothelial migration ability and other immunological functions. Furthermore, research on the interactions between NPs and platelets focuses on the activation degree and coagulation function, with relatively little attention to the structural changes in platelets themselves, which is why it is difficult to gain insights into the damage inflicted on platelets. Therefore, further research in this area is necessary.

In summary, this article focuses on the impact of NPs on platelet structure and function by delving into the mechanisms underlying their influence. These findings and trends can be summarized as follows. First, some NPs, such as chitosan, amorphous SiO_2_ NPs, and polystyrene NPs, can interact with platelet surface glycoprotein receptors, enhancing the interaction between fibrinogen and GPIIb/IIIa, and accelerate platelet activation, adhesion, and granule exocytosis. Related factors include the morphology, charge, concentration, and size of NPs. Second, NPs such as polyvinyl alcohol-coated superparamagnetic iron oxide NPs, amorphous SiO2 NPs, and polystyrene NPs can inhibit platelet activation by altering the conformational changes in fibrinogen. This alteration results in the failure of bridging between platelets, thereby slowing down platelet aggregation. Moreover, small-sized and negatively-charged polyethylene glycolized dendrimer NPs with relative low molecular weight and low concentration are more conducive to the stability and survival rate of platelets during storage. Thus far, we propose that nanomedical research would benefit from the proper use of nano-carriers with more understanding of the interaction between them and blood cells, including platelets, in the in vivo environment, to mitigate potential unforeseen complications during clinical trials.

This review can provide reference for exploring the relationship between the physicochemical properties and biological effects of nanomaterials. It may also help to enhance the in vivo safety of NPs and offer guidance for the development of novel nanomedicines or drug carriers.

## Figures and Tables

**Table 1:** Effects and interactions of different nanomaterials on platelet coagulation. NP: nanoparticle.

Material	Size (nm)	Effects on Platelets	Interactions with Platelets	Reference
Amorphous SiO_2_ NPs	10, 50, 150, 500	Accelerated aggregation	GPIIb/IIIa and P-selectin were exposed on platelet surface, and ADP ^1^ and MMP-2 ^2^ were released	[8]
Amorphous SiO_2_ NPs	70	Accelerated aggregation	Interaction with coagulation factor XII	[9]
Adenosine diphosphate Coated chitosan NPs	251.0 ± 9.8	Accelerated aggregation	Produces stronger clots in less time	[10]
Polystyrene NPs	25, 50, 119, 151, 201	Accelerated aggregation	Cause GPIIIb/IIIa exposure through passive aggregation and regulation by Src and Syk tyrosine kinases	[11]
Platinum NPs	7, 73	Accelerated aggregation		
Polystyrene latex NPs	50, 100	Accelerated aggregation	Endocytosis of the platelets and physical bridging to adjacent inactive platelets	[12]
Platelet-derived microparticles	100	Accelerated aggregation	Surface phosphatidylserine interacts with coagulation factors in plasma	[13]
ZnO NPs	20, 100	Inhibited aggregation	Reduced the thrombin generation potential	[5]
Ag NPs	13, 20, 29	Inhibited aggregation	Endocytosis of the platelets and inhibition of GPIIb/IIIa expression	[6]
Polyethylene glycol-coated silver NPs	20	Inhibited aggregation	Inhibits the synthesis of arachidonic acid into thromboxane and prevents GPIIb/IIIa exposure	[14]
Perfluorotributylamine NPs	150, 200	Inhibited aggregation		[15]
Polyvinyl alcohol-coated superparamagnetic iron oxide NPs	78 ± 22	Inhibited aggregation	Conformation of fibrinogen changes, causing the bridge between platelets to fail	[16]

^1^, ADP: adenosine diphosphate; ^2^, MMP-2: matrix metalloproteinase-2.

## Data Availability

Data is contained within the article.
